# A protocol for a randomized comparison of extended-release versus sublingual buprenorphine among pre-trial detainees in jail

**DOI:** 10.1186/s13722-025-00611-9

**Published:** 2025-10-27

**Authors:** Rebecca E. Rottapel, Thomas J. Stopka, Peter D. Friedmann, Randall A. Hoskinson Jr., Daviana Englander, Nicole Calhoun, Thomas Senst, Christopher Gudas, Peter J. Koutoujian, David Farabee

**Affiliations:** 1 Department of Public Health and Community Medicine, Tufts University School of Medicine, 136 Harrison Avenue, Boston, MA 02111 USA; 2 Department of Quantitative and Population Health Sciences, UMass Chan Medical School, Worcester, MA USA; 3 UMass Amherst, Amherst, USA; 4 Department of Population Health, New York University Grossman School of Medicine, 180 Madison Ave, 17th Floor, New York, NY 10016 USA; 5 Middlesex Jail and House of Correction, 269 Treble Cove Road, Billerica, MA 01862 USA

**Keywords:** Extended-release buprenorphine, Jail, Corrections, Massachusetts, Medication for opioid use disorder, Pre-trial

## Abstract

**Context/background:**

The high prevalence of opioid use among jailed adults offers an unmatched opportunity to identify and treat those with opioid use disorder (OUD), a population that is at a substantial risk for post-release overdose. From a public health perspective, jails are critical touchpoints, as these facilities typically admit more than 7 million adults per year. One clinical consideration is whether pre-trial detainees with OUD would benefit from early induction onto extended-release buprenorphine (XRB).

**Methods/study design:**

In this 3-year randomized controlled trial, we will identify and recruit 200 incarcerated adults with OUD who are receiving sublingual buprenorphine (SLB) or tolerate a SLB test dose and randomize them to receive extended release buprenorphine (XRB) (*n* = 100) or to remain on SLB (*n* = 100) while in custody. Study participation will continue through their pre-trial time in jail (up to 6 months) or until they are sentenced or released. Community treatment will then be tracked for 90 days following release. In addition to collecting data on XRB uptake in jail, we will assess (1) the percentage of XRB and SLB study participants leaving jail with a clinically active dose of buprenorphine in their system, (2) 90- day post-release MOUD continuation, (3) levels of buprenorphine diversion while in custody, and (4) recidivism and death (90 days). “Clinically active” is defined as receiving XRB within the past 28 days or SLB in the past 24 h.

**Discussion:**

Findings from this study will demonstrate the feasibility and outcomes of inducting pre-trial adults with OUD onto XRB, as well as offer practical clinical and policy guidelines for best practices for treating this high risk and understudied population.

## Introduction

### Context/background

Due to the high prevalence of opioid use and elevated risk of overdose among justice involved adults, jails offer an unmatched opportunity to identify and offer treatment to those with opioid use disorder (OUD). From a public health perspective, jails are critical intervention touchpoints, as these facilities typically admit more than 7 million adults per year in the US, holding individuals for an average of 32 days prior to release [1]. In Massachusetts, individuals released from incarceration have a 120 times higher rate of opioid-related overdose deaths compared to the remainder of the adult population [2]. Some jails have responded to the opioid crisis by expanding access to medication for opioid use disorder (MOUD) [3]. However, the optimal strategies for providing MOUD in jails—such as the most appropriate type and timing of treatment—are not well established.

One clinical consideration is whether XRB should be offered to pre-trial detainees with OUD. Relative to its more commonly used counterpart—daily SLB—XRB offers several potential advantages in jail, including: easier logistics of administration, reduced risk of diversion, reduced experiences of patient stigma due to the less regular dosing schedule, and an increased bridge of protection between the time of discharge and admission to community-based MOUD [4]. In prior analyses in Massachusetts jails, it was found that typical SLB retention rates upon release to the community were 80.6% for those prescribed SLB and 86.7% for those prescribed methadone within 30-days of release [5] (Personal communication), and a number of barriers to community connections and ongoing MOUD treatment continuity could lead to breaks in continued buprenorphine and methadone treatment [6, 7] (Stopka et al., 2024; Matsumoto et al., 2023). Because the release date for pre-trial detainees is variable and uncertain, early induction could ensure that persons released precipitously (e.g., from court without notice) will have a clinically active dose of buprenorphine in their system to protect them against post-release overdose. Early induction onto XRB is also more expensive than SLB.

The relative benefits of XRB versus SLB for patients in jail have yet to be established empirically, but a jail-based pilot study provides some reason for optimism [8]. A randomized comparison of 52 sentenced participants (half receiving XRB; half SLB) found that patients receiving XRB had fewer jail medical visits compared to those with daily SLB medication administration, higher enrollment in post-release community treatment (69% vs. 35%), and higher rates of opioid-negative urine tests following release (55% vs. 38%). These findings are promising and merit replication with a larger sample. It would be further beneficial to replicate that study with pre-trial detainees rather than sentenced residents, as the former account for more than 70% of the U.S. jail population [1], and are at greater risk for discontinued treatment and overdose given limited time for pre-release planning. In this study, 200 pre-trial detainees receiving SLB in a Massachusetts jail will be randomized to SLB treatment or transition to XRB treatment.

### Differences between SLB and XRB

SLB is a partial opioid agonist that is administered orally on a daily basis to those with OUD. The medication typically comes in a pill, tablet, or film, which is dissolved under the tongue. SLB dosages start at 2 mg and can be increased in 2 mg increments as deemed appropriate by medical and nursing staff. XRB is an extended-release version of buprenorphine and is indicated for the treatment of moderate to severe OUD in patients. XRB is a monthly injectable medication which is delivered subcutaneously to the abdomen, thigh, buttock, or back of the upper arm. A depot is formed at the injection site, which slowly releases the medication into the body. The XRB dose starts at 300 mg injection for the first two months, usually followed by a maintenance dose of 100 mg monthly thereafter depending on the clinical insights of the prescribing provider.

## Methods

### Overview of study design

This three-year research project will recruit 200 participants at the Middlesex Jail and House of Correction (MJHOC). The two-group randomized trial will assess the potential benefits of offering XRB to pre-trial detainees. Participating pre-trial detainees receiving SLB will be randomized 1:1 to transition to XRB as soon as possible or remain on SLB during their jail term (or up to six months). Per usual treatment for MOUD recipients at the jail, all efforts will be made to refer participants to receive MOUD treatment in the community upon release. We will compare (1) the percentages of XRB and SLB participants leaving jail with a clinically active dose of buprenorphine in their system, (2) infractions for diverting buprenorphine while incarcerated, (3) community MOUD intake and retention, and (4) 90-day recidivism or death. The final three months of the study will be devoted to data cleaning and analysis, study close out, preparation of manuscripts, and dissemination.

### Study population

Potential study participants will be pre-screened and then approached by research staff. If the participant is interested, they will undergo final screening and will be invited to complete the informed consent process. The screening criteria are described below.

#### Inclusion criteria

1. Incarcerated pre-trial detainees are able to provide written informed consent in English;
2. They are currently receiving SLB, which will be verified via the jail’s electronic medical system and medical staff, or have confirmed current OUD and tolerate a SLB test dose;
3. They are willing to accept random assignment to the experimental or control condition (i.e., transitioning to XRB while incarcerated or continuing on SLB).

#### Exclusion criteria

1. Sentenced detainees;
2. Based on charges, the anticipated jail stay is less than 4 days (e.g. larceny, license suspended, shoplifting, vandalize property). The estimated typical jail stay times will be made based on charges verified using the jail management system.

#### Participant recruitment

Following pre-screening, trained research staff will approach potential participants and inform them about study participation. Individuals who meet eligibility criteria and state clear interest in study participation will be invited to voluntarily complete the informed consent process. To ensure privacy, all pre-screening, screening, consenting, and interviews will take place in a private space, such as a medical exam room or space designated for attorney visits, or a private space on the unit. To further enhance confidentiality, the research staff will receive clearance at the jails as contractors so they will not need to disclose information to correctional staff when accessing prospective or enrolled participants’ information via the electronic medical records system or the jail offender management system.

**Fig. 1:**
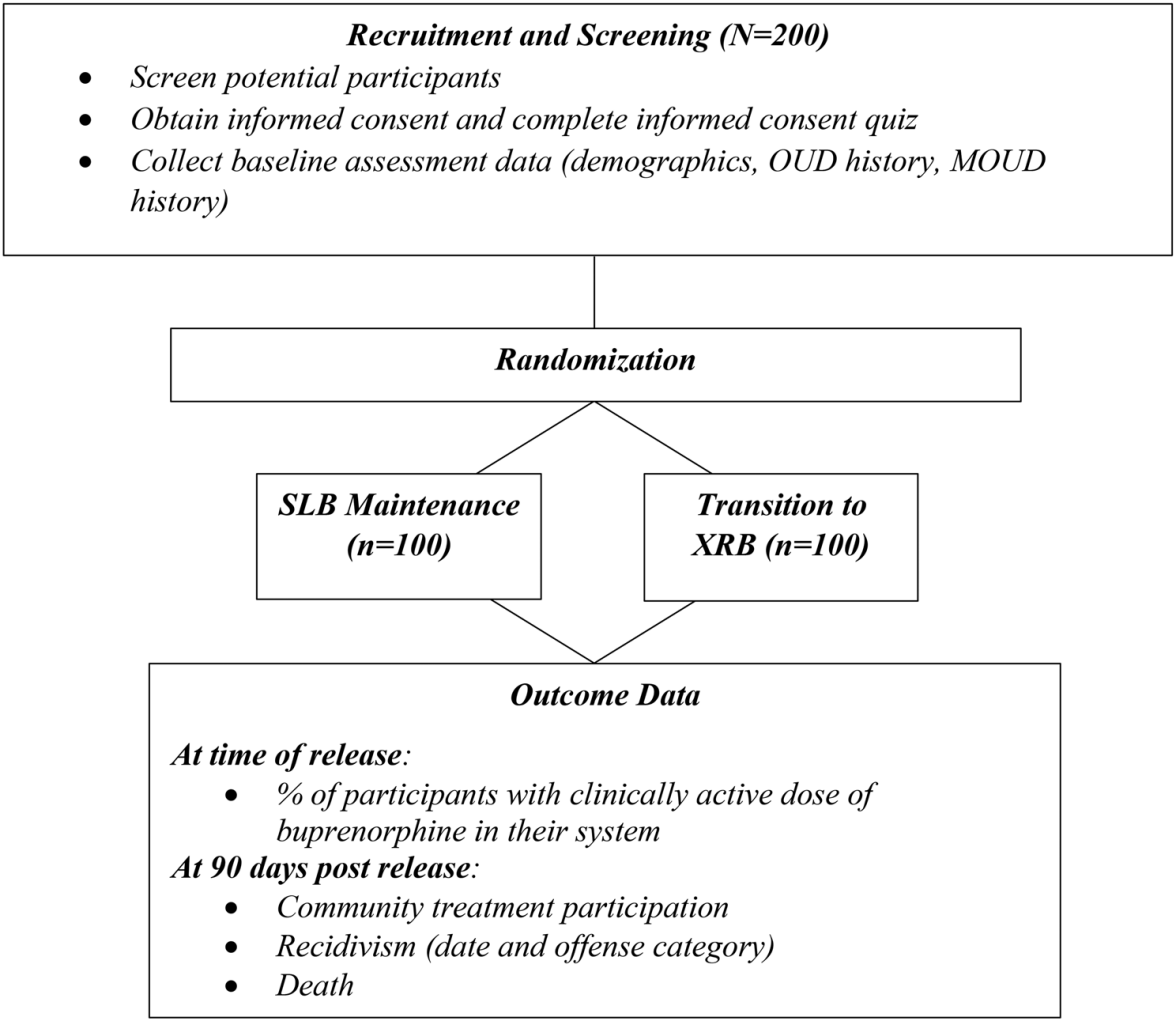
Study flow

Enrollment must be voluntary, and all participants can expect to benefit, in keeping with Office for Human Research Protections (OHRP) federal prisoner research standards. Enrollment status and study data or results will not be shared with judicial or correctional authorities, unless specifically authorized by the participant (Fig. 1).

### Treatment-as-usual procedures for jail inmates

Treatment-as-usual at MJHOC is provided through the Medication Assisted Treatment and Directed Opioid Recovery (MATADOR) program. MATADOR is a voluntary treatment option for individuals who meet eligibility criteria. This program offers all three forms of Food and Drug Administration (FDA) approved medications to treat OUD: methadone, buprenorphine, and naltrexone in combination with counseling, behavioral health therapies, post-release navigation supports, and helps connect patients to treatment providers in the community post-release. Through this program, participants receive support services from MJHOC staff to work towards their recovery and develop a long-term individualized treatment plan. Additional support from MJHOC Patient Navigators (PNs) and Recovery Coaches is available to help with transitioning back to the community post-release. This is the standard of care for all MOUD patients at the jail.

Recruitment targets were based on numbers provided by the MATADOR program indicating that between January 2024 and March 2025, MSO enrolled on average 16 pre-trial patients per month who were receiving daily buprenorphine, with a range of 8–26 enrollments and a median of 16. In addition, we plan to expand enrollment to pre-trial incarcerated adults on SLB who are not recent admissions but are within 90 days of anticipated release.

### Study sites

The site for participant recruitment, data collection, and treatment will be the MJHOC. Middlesex is the most populous county in Massachusetts and New England [9]. The facility includes a House of Correction, which holds people who are sentenced, and a jail, which holds people awaiting trial. As of June of 2025, 526 pre-trial detainees were being held at MJHOC [Farabee D, 2024, 2 Questions, personal communication]. This study will only include unsentenced, pre-trial detainees (Table 1).

**Table 1:** Data sources and measures

Measures	Data source	Screening	Pre-release	Post-release
Screening (adult, SLB, unsentenced, 4 days-6 months anticipated jail stay, willing to be randomized)	Electronic medical record (CorEMR), Offender Management System (OMS), self-report	X		
Baseline	Self-report		X	
* Demographics*				
* OUD history*				
* MOUD history*				
Dosing schedule (SLB and XRB)	CorEMR		X	
Liver function test (LFT)	CorEMR		X	
Urine drug screening	CorEMR		X	
Medication diversion incidents	OMS and Inner Perimeter Security (IPS) unit		X	
Number of days Buprenorphine protection post-release (7 days)	CorEMR, OMS			X
Enrollment in community based MOUD (90 days)	Community clinics			X
* # of appointments*				
* Medication type*				
* Dose amount*				
Recidivism (90 days)	Criminal Justice Information Services (CJIS)			X
* # of arraignments*				
* Types of charges/offenses*				
* # of incarcerations*				
* # of convictions*				
Death (90 days)	Various sources (Massachusetts Office of vital statistics, National death index, Google)			X

### Outcome measures

The study is assessing the impact of offering XRB to pre-trial incarcerated adults, as compared to those maintained on SLB (treatment as usual). Descriptive statistics will provide an overall assessment of the implementation of the protocol.

#### Primary outcome measure

The primary outcome measure is the percentage of participants who leave with a clinically active dose of buprenorphine in their system.

#### Secondary and tertiary outcome measures

The secondary outcome measure is MOUD continuation in the community. This outcome will be based on community clinic records indicating admission and retention at 90-days following release, measured as the number of SLB doses or XRB injections received in the community. Tertiary outcome measures involve a range of incidents including death, diversion, disciplinary actions, as well as recidivism outcomes at 90 days post release. Recidivism will be defined as arraignments, types of charges/offenses, incarcerations, and convictions. Data will be acquired through the jail from Criminal Justice Information Services (CJIS) records.

### Study procedures

#### Administrative approvals

The NYU Langone Health Institutional Review Board (IRB), which serves as the single IRB, reviewed and approved the study with the Middlesex County Sheriff’s Office and the Tufts University School of Medicine. To further protect the privacy of study participants, the National Institutes of Health (NIH) issued a Certificate of Confidentiality. In addition, given that this study includes prisoner involvement, approval was received from the Office of Human Research Protections (OHRP). The study has been registered on clinicaltrials.gov (NCT05481112).

The trial will be carried out in accordance with International Conference on Harmonization Good Clinical Practice (ICH GCP) and the following: (1) United States (US) Code of Federal Regulations (CFR) applicable to clinical studies (45 CFR Part 46, 21 CFR Part 50, 21 CFR Part 56, 21 CFR Part 312, and/or 21 CFR Part 812), (2) All key personnel and clinical trial site staff who are responsible for the conduct, management, or oversight of this trial will complete Human Subjects Protection and ICH GCP Training.

#### Screening

Research staff will review jail records to identify potentially eligible participants. Thereafter, they will arrange to meet individuals in person to share preliminary information about the study and, if interested, complete the in-person screening process. This process will include asking if they are willing to accept randomization to the experimental or enhanced usual care condition and ensuring that they are able to provide written informed consent in English. Eligible and interested participants will be invited to begin the consent process.

#### The consent process

Consent forms will describe, in detail, the study intervention, study procedures, and risks. Informed consent is required prior to starting the study intervention. Participants will be provided with a copy of the informed consent form. Trained research staff will review the consent form making sure to explain the study including a review of the study purpose, procedures, potential risks and of the rights of research participants. Participants will be informed that participation is voluntary and that they may withdraw from the study at any time, without prejudice, nor impacts on their ongoing services received. They will also be notified that a Federal Certificate of Confidentiality has been obtained to encompass protocol activity and further protect confidential study participation. Staff will answer any questions that may arise.

This conversation will take place in a private room or private area on the unit. Participants will have the opportunity to carefully review the written consent form and ask questions prior to signing. The participant will be offered the opportunity to discuss the study with anyone they so choose or take time to think about their choice prior to agreeing to participate. If they choose to participate, the participant will sign the informed consent document. A copy of the informed consent document will be given to the participants for their records.

#### Random assignment

Randomization of eligible participants will occur within one week after screening. A randomization module has been created on REDCap, using a 1:1 permuted block design. Study assignment is unblinded. Participants and medical staff will be notified of the study arm assignment. It is anticipated that screening, the consent process, randomization, and treatment induction will likely occur within a 5-day window. While randomization to the experimental condition will not guarantee XRB induction, every practical effort will be made to induct participants onto XRB prior to release. It is, however, the nature of the unsentenced population that release dates are unpredictable. The percentage of participants in the experimental condition who do initiate XRB prior to release will be an important implementation finding of this project.

##### SLB

As described above, participants will already be receiving SLB and be participating in the MATADOR program. If randomized to the SLB arm, they will continue treatment as usual in the facility.

##### XRB

The Sublocade packaging states that patients with OUD who are not on an active SLB dose may receive XRB if they are able to tolerate a 4 mg dose. If this dose is tolerated for an hour without precipitated withdrawal, the initial monthly injection can be administrated. As previously mentioned, XRB injections start at 300 mg for the first 2 months, usually followed by monthly maintenance doses of 100 mg. Study clinical staff will have flexibility to continue the 300 mg dose for greater than two months, or use the 100 mg dose for initial induction, based on FDA-labeling for XRB dosing, if the participant’s opioid use history or clinical status at the time of dosing support these decisions. Medication induction and maintenance will be conducted in accordance with the FDA-approved labeling and associated guidelines.

Jail medical staff will be encouraged to deliver XRB in a manner consistent with the FDA label and this protocol, but also in a flexible, pragmatic paradigm given staffing, internal policies, clinical settings, additional counseling resources, and general medical and psychiatric care at MJHOC, which may impact and interact with study treatment. (Based on the study physician’s experience, most XRB participants are expected to remain on the higher dose.) This protocol explicitly welcomes and tolerates these types of real-world factors, and potential confounders, consistent with an open label effectiveness trial or pragmatic trial. Medication treatment is free-of-charge but not otherwise incentivized.

##### Dosing timelines and release

In both SLB and XRB conditions, patients will be encouraged to continue their treatment outside of the facility. Both forms of treatment that are offered in the study are available to participants in the community. Designated MATADOR staff at the MJHOC create appointments for patients to continue receiving their medication in the community. These appointments are set up prior to release, however it is the patients’ responsibility to follow through with these appointments and continue their treatment. Patients are also given a bridge script for their medication prior to discharge in attempt to avoid lapses in treatment.

##### XRB injection procedures

###### XRB induction

Patients randomized to the XRB condition will first receive a 4 mg dose of sublingual buprenorphine followed by the XRB injection an hour later, if there is no precipitated withdrawal, based on package insert recommendations. XRB will be delivered as a pre-filled 2 cc subcutaneous monthly injection, using a 300 mg starting dose in most cases. Study staff may elect to use the 100 mg dose for initial injection, if over-sedation is deemed to be a higher-than-average risk (for instance, if the participant experienced over-sedation during the SLB up-dosing). XRB consists of a depot injectable formulation in polymeric solution to the abdomen and releases buprenorphine over 28-days (4-weeks) by diffusion as the polymer biodegrades.

###### XRB maintenance

Participants will receive XRB monthly from the time of induction to the day of release, sentencing, or transfer. Study clinicians will generally adhere to FDA-labeling on XRB dosing, which recommends two months of 300 mg doses followed by maintenance doses of 100 mg monthly for the remaining four months. The maintenance dose may be increased to 300 mg monthly for patients who tolerate the 100 mg dose, but do not demonstrate a satisfactory clinical response, as evidenced by self-reported illicit opioid use or urine drug screens positive for illicit opioid use. Indivior data indicates that 300 mg continuous dosing may be more effective for people experiencing fentanyl-involved OUD. XRB can be administered subcutaneously in the abdomen, thigh, buttock, or back of the upper arm at each 28-day visit with a minimum of 26 days between doses. In the event of a missed visit, the dose can be delivered within 28 days (up to 8 weeks since the prior dose). Longer missed dose windows will require another brief SLB lead-in prior to the next injection.

#### Study medication storage

Each single XRB dose is packaged in a carton in a prefilled syringe with safety needle. It is stored in refrigerator at 2–8 °C (35.6–46.4 °F). Once outside the refrigerator this product may be stored in its original packaging at room temperature, 15–30 °C (59–86 °F), for up to seven days prior to administration. The product requires at least 15 min to reach room temperature.

#### Dispensing of study medications

After administration, syringes should be properly disposed, per procedure for a Schedule III drug product, and per applicable federal, state and local regulations. If the XRB dose is left at room temperature for longer than seven days, it should be discarded.

#### Enhanced treatment-as-usual: patient navigator

All study participants will be given access to enhanced patient navigation, beyond what is currently offered by the jail (a single follow-up immediately following release). The study will provide an enhanced PN to facilitate their return to the community and to encourage continued participation in MOUD treatment. This approach has shown promising effects for adults leaving prison [10]. The PN from this project will collect locator data from the participant prior to release and attempt to contact the participant at 30, 60, and 90 days after the discharge date. The focus of the PN will be to (1) ask if there are any re-entry barriers that need to be addressed, and (2) encourage ongoing MOUD engagement in the community. The PN will endeavor to reach all participants at least one-time at each follow-up timepoint and will make follow-up contacts as is relevant.

#### Participant withdrawal

Participants are free to withdraw from the study at any time upon verbal request. An investigator may discontinue or withdraw a participant from the study if any clinical adverse event (AE), laboratory abnormality, or other medical condition or situation occurs such that continued participation in the study would not be in the best interest of the participant.

Subjects who sign the informed consent form and are randomized but are not assigned to a study condition (i.e., initiate/continue treatment relevant to their study arm) may be replaced. Subjects who sign the informed consent form and are randomized and successfully assigned to a study condition, and subsequently discontinue from the study, will not be replaced.

Participants in either treatment group that choose to drop out of the study during their incarceration will be transitioned back to the standard of care at facility. Participants who drop out from the study will no longer be contacted, receive patient navigation services, or be followed for study purposes.

#### Subject payments

The study will provide non-coercive monetary incentives to participants. Participants will receive a letter, placed among their personal belongings that will be returned upon release from MJHOC, with the study’s contact information and be paid retroactively ($40). Following release, the participant will contact study staff who will coordinate the provision of payment to the participant.

## Results/hypotheses

### Planned analyses

The approach for testing the primary hypothesis— that administering XRB to pretrial detainees will result in a significantly higher proportion of participants being released with a clinically active dose of buprenorphine in their system than will SLB (TAU) participants— will use generalized linear models with a log-binomial link to generate risk ratios for the study groups. Outcome measures for the secondary outcomes include rates of enrollment in community MOUD following release from jail. We will also assess group differences in the infractions for diversion and recidivism. Differences across treatment arms (e.g., disciplinary infractions) will be assessed using generalized linear models depending on the nature of the outcome. In the case where there are repeated measurements for each individual (e.g., buprenorphine treatment continuity at X, Y, Z months), mixed effect models will be used with participant-level random effects.

This study is powered to detect a medium effect size corresponding to a risk ratio of 1.34 (OR = 2.4), assuming 75% are released from jail with a clinically active dose of buprenorphine in their system in the XRB condition, a two-tailed test, and a power of 0.80. Because the desired outcome is expected to be rare under the SLB condition, we also consider the precision of the primary outcome prevalence estimate under the XBR condition given the total sample size (*n* = 200). With *n* = 100 participants in the XBR condition and 0.75 expected for the prevalence of the primary outcome, the 95% confidence interval width is 0.168 (i.e., 0.657–0.825).

Retention-in-study-medication-treatment will be tracked using administrative data. We will make every attempt to confirm suspected deaths, cause-of-death, and relevant collateral data through participant contacts, public information, and public death data.

We will use a modified intention-to-treat (mITT) approach. The primary analysis sample will be participants randomized and released to the community. Clinical trials in the criminal legal system usually see a small rate of randomized participants initially planning for release, then sentenced, transferred, or otherwise incarcerated for longer periods of time due to unanticipated post-randomization factors independent of the study. This effectively renders the now randomized participant ineligible, as they are unable to leave the jail and continue treatment in the community. Thus, participants not released to the community as planned will be excluded from the analysis of the primary outcome. Participants who are not released to the community do not enter the risk set for outcomes that can only be observed at release or in the community after release. While we do not expect arm differences in the probability of release to the community, we will examine this as an exploratory outcome descriptively and with a Fisher’s exact test in intent-to-treat (ITT) analysis. In other ITT analyses, the approach will be outcome-dependent. For the outcomes of buprenorphine in a participant’s system at release and participation in MOUD treatment following release, these participants would be coded as No. For buprenorphine diversion during incarceration, these cases need no special treatment since the outcome does not depend on release. For rearrests, ITT analysis would code these participants as not rearrested. In all modified intent-to-treat (mITT) or per protocol analysis of outcomes observed at or after release to the community, participants not released to the community will be excluded. We expect this to occur among fewer than 5% of all participants who are randomized. Secondary analyses will examine all randomized participants (ITT) and participants both inducted and released as planned (per protocol). Sensitivity analyses will examine the effect of competing risks (i.e. incarceration or death) after release on the primary outcomes.

## Discussion/conclusions

Given the high prevalence of opioid use among adults involved with the criminal legal system in the United States, it is critical to identify and treat jail residents who meet the criteria for OUD. Although most jail-based studies have focused on initiating MOUD for sentenced residents nearing release, this study targets pre-trial residents. Those under pre-trial status are generally more difficult to treat with XRB, as their uncertain discharge dates reduce providers’ confidence in completing the weeklong induction phase. But successfully initiating XRB for this population is likely to increase the rate of participants leaving jail with a clinically active dose of buprenorphine in their system and reduce their risk of opioid-related relapse and overdose during the high-risk phase immediately following release from jail. Findings from this study will demonstrate the feasibility and outcomes of inducting pre-trial adults with OUD onto XRB, as well as offer practical clinical and policy guidelines on how best to do so. Final outcomes are expected by spring of 2027.

## Data Availability

No datasets were generated or analysed during the current study.
